# 80 Hz but not 40 Hz, transcranial alternating current stimulation of 80 Hz over right intraparietal sulcus increases visuospatial working memory capacity

**DOI:** 10.1038/s41598-022-17965-8

**Published:** 2022-08-12

**Authors:** Jimin Park, Chany Lee, Sangjun Lee, Chang-Hwan Im

**Affiliations:** 1 Department of Biomedical Engineering, Hanyang University, 222 Wangsimni-ro, Seongdong-gu, Seoul, 04763 Republic of Korea; 2 Department of Electronic Engineering, Hanyang University, Seoul, Republic of Korea; 3 Department of Structure & Function of Neural Network, Korea Brain Research Institute, Daegu, Republic of Korea

**Keywords:** Working memory, Biomedical engineering

## Abstract

Working memory (WM) is a complex cognitive function involved in the temporary storage and manipulation of information, which has been one of the target cognitive functions to be restored in neurorehabilitation. WM capacity is known to be proportional to the number of gamma cycles nested in a single theta cycle. Therefore, gamma-band transcranial alternating current stimulation (tACS) should be dependent of the stimulation frequency; however, the results of previous studies that employed 40 Hz tACS have not been consistent. The optimal locations and injection currents of multiple scalp electrodes were determined based on numerical simulations of electric field. Experiments were conducted with 20 healthy participants. The order of three stimulation conditions (40 Hz tACS, 80 Hz tACS, and sham stimulation) were randomized but counterbalanced. Visual hemifield-specific visual WM capacity was assessed using a delayed visual match to the sample task. High gamma tACS significantly increased WM capacity, while low gamma tACS had no significant effect. Notably, 80 Hz tACS increased WM capacity on both the left and right visual hemifields, while previous tACS studies only reported the effects of tACS on contralateral hemifields. This is the first study to investigate the frequency-dependent effect of gamma-band tACS on WM capacity. Our findings also suggest that high gamma tACS might influence not only WM capacity but also communication between interhemispheric cortical regions. It is expected that high gamma tACS could be a promising neurorehabilitation method to enhance higher-order cognitive functions with similar mechanisms.

## Introduction

Working memory (WM) is an essential human cognitive function that allows for the temporary storage and manipulation of information in various goal-directed behaviors^1,2^. Because both storage and manipulation are inherent in the definition of WM, most established WM models for analyzing WM capacity account for both factors^3,4^. Traditionally, the neural substrate of WM capacity has been studied with the aid of various brain imaging technologies, such as electroencephalography (EEG) and functional magnetic resonance imaging (fMRI); however, recent developments in non-invasive brain stimulation (NIBS) technologies have allowed for the investigation of alternation of WM capacity in response to currents delivered transcranially, by modulating neuronal features according to various properties of the injection currents.

Among the various NBS methods, transcranial alternating current stimulation (tACS) has drawn increased attention as a useful tool for modulating endogenous brain oscillations. While transcranial direct current stimulation (tDCS) or repetitive transcranial magnetic stimulation (rTMS) affects the excitability of cortical neurons, tACS is known to entrain brain oscillations to alternating current that were externally delivered^5,6^. It is widely believed that an external sinusoidal AC entrains neuronal oscillatory activity to the current, thereby modulating the brain functions associated with specific oscillatory activity. Nonetheless, the effectiveness of tACS is known to be highly dependent on the frequency of the injection current, as reported in multiple studies that used EEG recordings^5,7,8^ and behavioral measures^9–12^.

Multiple attempts have also been made to modulate WM capacity using tACS at different stimulation frequencies^12–14^. Notably, it has been reported that controlling the speed of the theta band (4–7 Hz) could either increase or decrease WM capacity^12^. The reason why slow and fast theta band tACS have differential effects on WM capacity can be explained by the relationship between the theta-gamma cross-frequency coupling (CFC) of brain waves and WM capacity, which is thought to be proportional to the number of gamma (> 40 Hz) cycles embedded in a single theta (4–7 Hz) cycle. Thus, the slower the theta wave and the faster the gamma wave, the larger the WM capacity, and vice versa^15,16^.

While gamma oscillation is another parameter that may influence the abovementioned CFC characteristics and WM capacity, previous studies on the effects of gamma tACS on WM performance did not report consistent outcomes. Indeed, gamma tACS has been reported to enhance WM performance in some studies^14^ but was not effective or consistent in other studies^17,18^. Notably, the stimulation frequencies used in previous studies were 40 Hz or lower. However, as mentioned above, the WM load has a positively linear relationship with the speed of the gamma oscillation. It is to be noted that the most commonly studied frequency, 40 Hz, is closer to the lower bound of the gamma-band. Therefore, to enhance WM with gamma tACS, it may be more appropriate to use a frequency higher than 40 Hz to increase the number of gamma cycles nested in a single theta cycle. Therefore, we compared the effects of 40 Hz and 80 Hz tACS on visuospatial working memory (VWM) by selectively stimulating the right intraparietal sulcus (rIPS). For focal stimulation, we used a multi-electrode setup, with optimal injection current amplitudes and electrode locations being found using the finite element method (FEM) and least squares (LS) algorithm. We hypothesized that VWM capacity would be enhanced more clearly by 80 Hz tACS than by 40 Hz tACS.

## Methods

### Participants

Twenty-five healthy young university students (17 males, 8 females; all right-handed) participated in this study. Individuals with any identifiable neurological disorder, head injury, or any personal or family history of psychiatric illness were screened prior to the start of the experiment. Participants were informed to refrain from intaking caffeine, nicotine, or alcohol within 12 h of the start of the experiment. Five participants who failed to participate in any of the three conditions were excluded from the analyses. The mean and standard deviation of the ages of the participants included in the analysis were 23.94 ± 2.02 years. Note that only one of the participants was a mild smoker. Written informed consent was obtained from all the participants prior to their participation. All experimental procedures were approved by the institutional review board (IRB) committee of Hanyang University, South Korea (IRB No. HYU-2019-07-008). All procedures, including the tasks and stimulations, were carried out in accordance with the Declaration of Helsinki.

### Experimental design

The participants participated in two active stimulation sessions (80 Hz and 40 Hz) and a sham stimulation session. The three sessions were randomly counterbalanced, and each session was at least 72 h apart to avoid potential carry-over effects. The sessions started at the same time on differnt days. During the stimulation, participants were asked to perform a VWM task.

The stimulation current was delivered using Starstim (stimulator) and Sponstim (circular electrode, area = 8 cm^2^) manufactured by Neuroelectrics (Barcelona, Spain). The electrodes were saline-soaked and placed over PO4, Pz, and T8 according to the international 10–20 EEG system. The peak to baseline amplitudes of the injection current were 1 mA, 0.64 mA, and 0.36 mA, respectively, for PO4, Pz, and T8 electrodes, as determined in simulations. The electrode montage and conditions were determined based on numerical simulations, which are further described in “Electric field simulation” and “Optimization” sections. All conditions for the active (80 Hz and 40 Hz) and sham stimulations included ramping-up and fading-out periods of 20 s each. During the ramping-up and fading-out periods in the sham stimulation condition, the stimulation frequency was set to 60 Hz. The stimulation frequencies of 40 and 80 Hz were selected because they were commonly used in previous gamma and high-gamma tACS studies^19–21^. Additionally, 60 Hz, the mean frequency, was selected for the sham stimulation to eliminate any potential influence of sham stimulation in the behavioral performance (see^12^ for selection of mean frequency as the frequency for sham condition).

Each stimulation session lasted 20 min, when no current was injected during the sham stimulation session after the consecutive ramping-up and fading-out periods. It was confirmed that participants were unaware of the different stimulation conditions.

### Behavioral task

**Figure 1:**
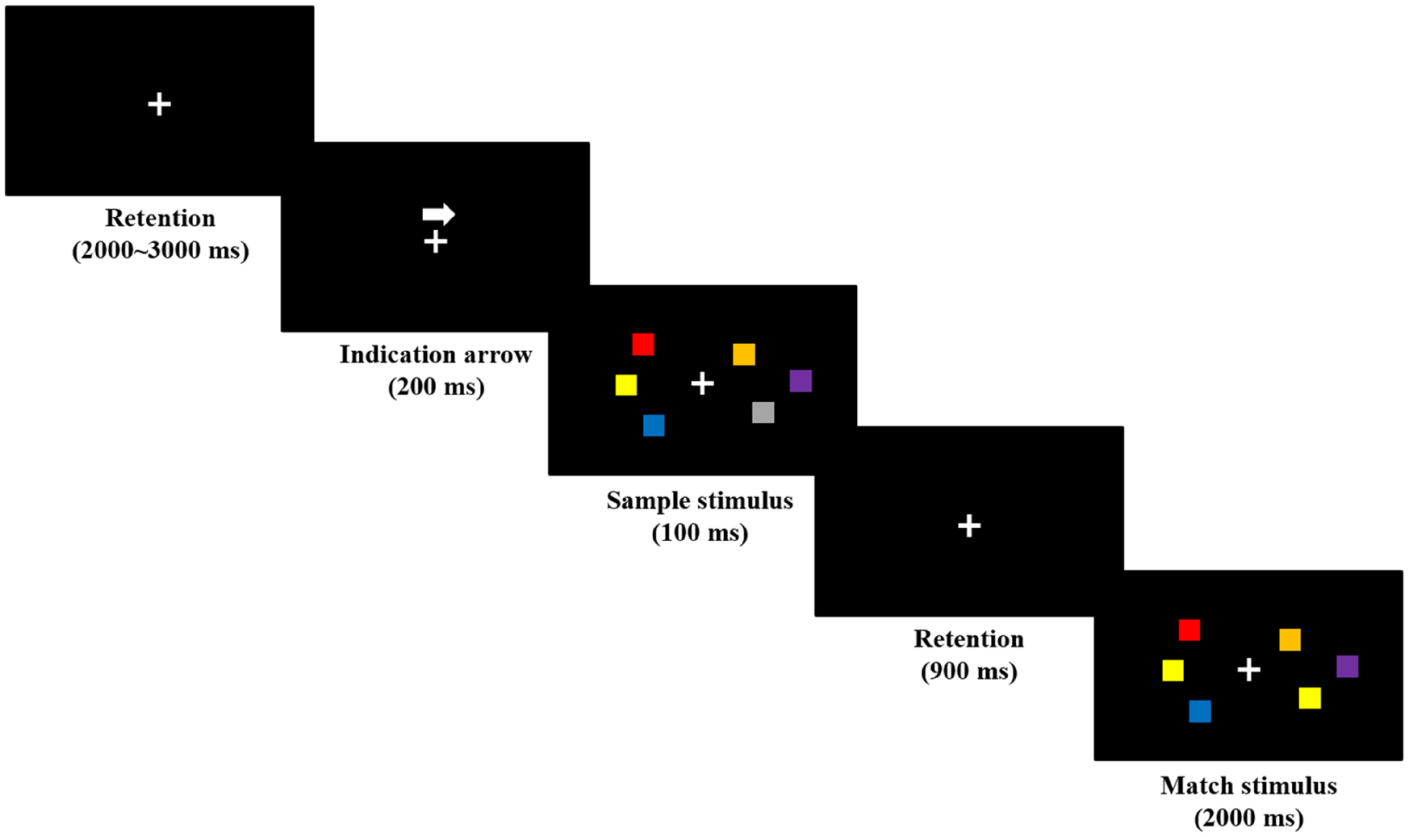
Flowchart of the visual match to delayed sample task. Two arrays of colored squares were presented on both sides of the fixation cross, with no square on the side sharing a color. The number of squares presented were four, five, and six on the real task (three on the figure). An indication arrow (200 ms) informing which side of the arrays were to be memorized preceded the sample stimulus (100 ms), followed by the retention period (900 ms) and match stimulus (2000 ms). When the match stimulus was presented, participants were asked to determine whether the arrays that the indication arrow pointed to have the same colors as the sample stimulus.

The VWM load was measured using the visual delayed match-to-sample task, which was mostly replicated from previous studies^12,22^. Figure 1 shows the graphical flowchart of the task. The task started with a fixation cross, which was displayed throughout the task procedure. An arrow pointing either left or right appeared for 200 ms above the fixation cross, followed by a 100 ms display of two arrays of squares with different colors on each side of the fixation cross. The arrow that appeared prior to the presentation of the square arrays allowed the participants to know which of the two arrays should be memorized. After the square arrays disappeared, only the fixation cross appeared on the screen for 900 ms, which was referred to as the retention period. After the retention period, another set of square arrays appeared on the screen for 2000 ms, as depicted in Fig. 1. The participants were then asked to determine whether the newly appeared square array in the same direction as the arrow orientation possessed the same colors as the firstly shown square array. During the display of the second square array, participants were asked to press the right button of the keyboard if the first and second arrays matched and the left button if they mismatched. The trials were separated into 2000–3000 ms random prestimulus retention periods, during which only the fixation cross was presented.

The entire task was separated into six blocks, each consisting of 20 trials, resulting in a total of 120 trials. The number of squares in an array of blocks, which represents the VWM load, was one of four, five, or six, with two blocks of each VWM load. For each visit, six blocks (two blocks per load) presented in the task were randomly chosen from nine preset blocks (three blocks per load). Each block consisted of five right-matched, five right-mismatched, five left-matched, and five left-mismatched trials, respectively. Participants were informed that they could take a break in between blocks, but no participant wanted to take a break during the task.

### Procedure

After the stimulation electrodes were set up, verbal instructions were provided to participants. After the instruction was given, a practice block with three VWM load was carried out. The load of the practice session was set to three to prevent any possible load-specific learning effects when performing the main session. After the practice block was completed, additional saline solution was added if necessary. The stimulation began, and the participants were instructed to start the first of the six main blocks; that is, the task blocks were performed simultaneously with the tACS. Once each block was finished, the participants could progress to the next block by pressing the Enter key on the keyboard. Approximately 13–14 min was required to complete all the main blocks.

### Data analysis

Rather than directly assessing the accuracy, the K-value was calculated using a formula suggested in a previous study^12,23^ to consider the load of the task. Because the loads were different for each block, using K-value better represents behavioral performance of WM task than directly analyzing the accuracy of all blocks. In addition, the reaction times were analyzed.

Because Kologorov-Smirnov test showed that the data did not follow normal distribution, a repeated measure of Friedman’s analysis of variance was performed for within-factors load (four, five, and six) and condition (80 Hz, 40 Hz, and sham). Both factors were analyzed separately for the left and right visual hemifields. If any of the Friedman’s tests reached a significant level (p < 0.05), Wilcoxon’s signed-rank test was carried out as the *post-hoc* test. Finally, the false discovery rate (FDR) was employed for multiple comparison correction if necessary.

### Electric field simulation

**Figure 2:**
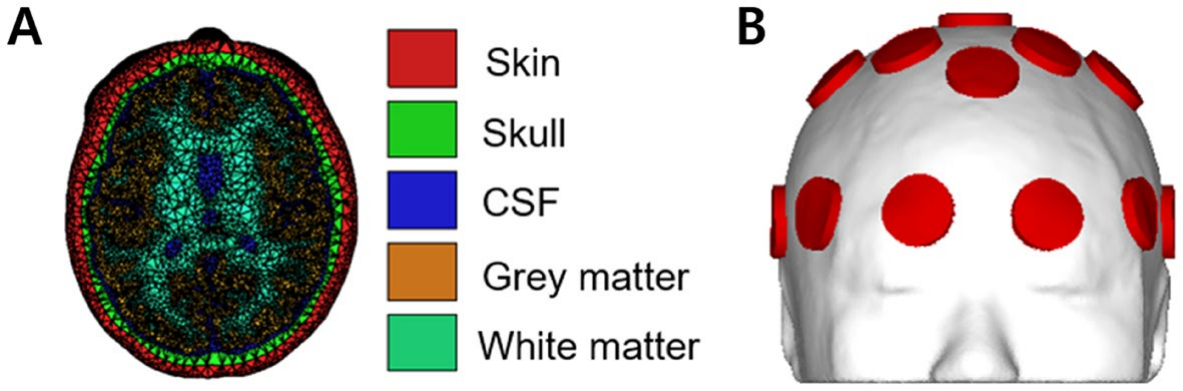
The model used for FEM calculation. (**A**) MRI-based realistic finite element model; consists of the skin, skull, cerebrospinal fluid, gray matter, and white matter, (**B**) scalp surface with circular electrodes with an area of 8 cm^2^ generated.

We conducted simulation and optimization study to focally stimulate the IPS for two reasons: (i) because the IPS covers wide range of parietal electrodes, and (ii) sulci are harder to deliver focal stimulation due to geometric features. The optimal electrode montage and injection currents were determined using a sample magnetic resonance image data. The electric field distribution over the cortex was computed using FEM, with a realistic finite element (FE) head model constructed based on the sample MRI data. The MRI dataset acquired using a 3 T MAGNETOM Trio Scanner (Siemens, Erlangen, Germany; spatial resolution: 0.8 mm × 0.8 mm × 0.8 mm). Sub-volumes of the head model, which included the scalp, skull, cerebrospinal fluid, gray matter, and white matter, were segmented using SimNIBS^24^ and itk-snap^25^. Circular electrodes with an area of 8 cm^2^ (equivalent to the area of Sponstim used in the main experiment) were generated over the scalp using an in-house script of MATLAB 2018a (MathWorks, Natick, MA, USA). The number and locations of the attached electrodes are further described in the “Optimization” section. The segmented FE head model and the model with electrodes are depicted in Fig. 2.

The FEM solver embedded in COMETS2^26^ was employed to solve the Laplace equation ${\nabla} \cdot \left({\sigma}{\nabla}{\text{V}}\right)= \text{0}$ (*V*: electric potential; *σ*: conductivity). Total of N-1 electric field distributions (N = the number of electrodes) were computed, as Dirichlet boundary conditions were imposed over the distal surfaces of “active” and ground electrodes, respectively. The boundary condition was scaled so that the current flowing into the ground electrode became 1 mA. In the calculation of electric fields, all tissues were assumed to be isotropic, with conductivity values of each tissue being set according to a previous study^27^.

### Optimization

**Figure 3:**
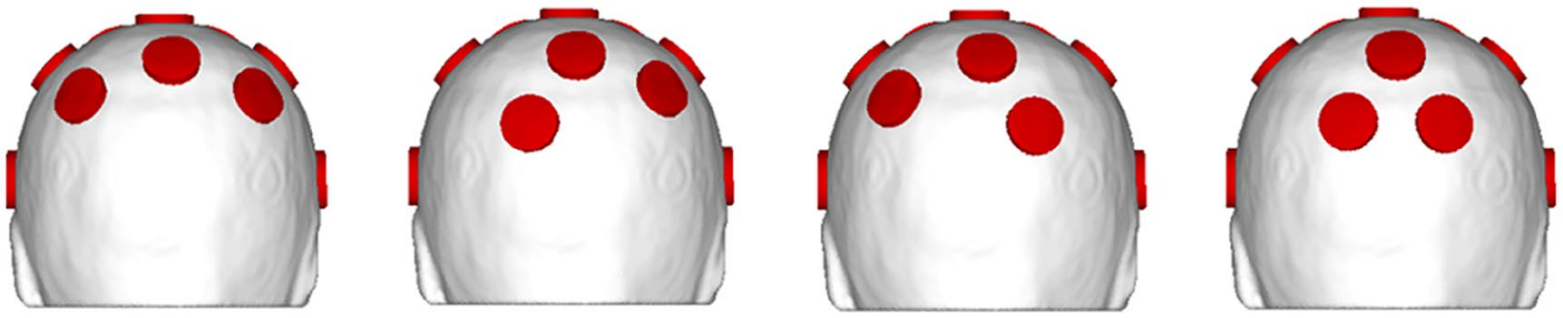
Four 15-channel montages used for channel selection. From left to right: P3-P4, PO3-P4, P3-PO4, PO3-PO4 configurations.

Fifteen electrodes were attached according to the Neoprin headcap (Neuroelectrics, Barcelona, Spain), which was developed based on the international 10–20 EEG electrode system. It is to be noted that only 15 electrodes could be attached without overlapping although 32 locations were available on the cap. Because change of electrode positions can significantly alter stimulated cortical regions, we tested four sets of different electrode locations to determine electrode locations that best stimulates the IPS. A 15-electrode montages were constructed, with the locations of 13 electrodes fixed and two electrodes attached at one of P3/PO3 and one of P4/PO4 position (see Fig. 3). A previous tACS study that targeted the right intraparietal sulcus (rIPS) placed 5 × 7 cm^2^ electrodes over P4 and the contralateral supraorbital area to deliver the electric field to the entirety of region of interest (ROI) ^12^; since we used considerably smaller electrodes, more precise electrode locations needed to be determined to effectively stimulate the entire ROI.

For each of the four montages, the LS algorithm was used to calculate the optimal injection currents to stimulate the rIPS. LS is an algorithm that determines the solution (injection currents in this case) by minimizing the error between the objective function and the actual solution. The objective function used in this study is as follows:

1 $$f\left( \phi \right) = \left\{ {\begin{array}{*{20}ll} 1 & \quad {if\;\Omega \; \in {\text{ROI}}} \\ 0 & \quad {if\;\Omega \; \notin {\text{ROI}}} \\ \end{array} } \right.,$$

where ROI indicates the region of interest, which was IPS0 and IPS1 of the rIPS.

Then, from the montage which induced maximum electric field over the ROI, three electrodes with the highest injection current amplitude were selected to determine the final three-electrode montage. Finally, LS was utilized again to determine the optimal injection current conditions.

### Ethics approval and consent to participate

All experimental procedures were approved by the institutional review board (IRB) committee of Hanyang University, South Korea (IRB No. HYU-2019-07-008). All participants agreed on written consent that is in accordance with the IRB approval before the experiment.

## Results

**Figure 4:**
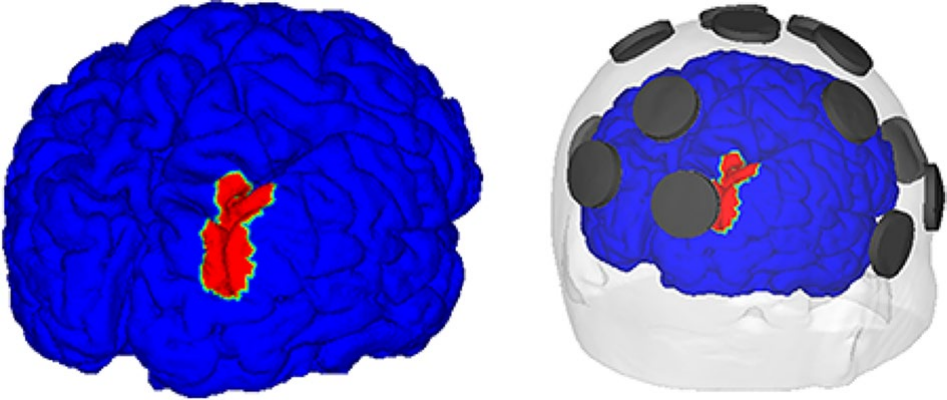
The ROI and an exemplary demonstration of electrode configuration relative to the ROI (PO3-PO4 montage, only for illustrational purpose).

According to the simulation results, the maximum electric fields over ROI were 0.19 V/m, 0.19 V/m, 0.23 V/m, and 0.22 V/m, respectively, for P3-P4, PO3-P4, P3-PO4, and PO3-PO4 montages (see Table 1). The ROI, electric field distribution, and optimal injection current conditions for each montage are shown in Fig. 4.

**Table 1:** Characteristics of electric field delivered and selected channels using 15 channel montages.

Montage	P3-P4	P3-PO4	PO3-P4	PO3-PO4
Emax_ROI_	0.194	0.232	0.191	0.225
Emax_NonROI_	0.2	0.227	0.192	0.239
Selected channels	Pz, P4, C4	PO4, Pz, T8	Pz, P4, C4	PO3, PO4, Pz

**Figure 5:**
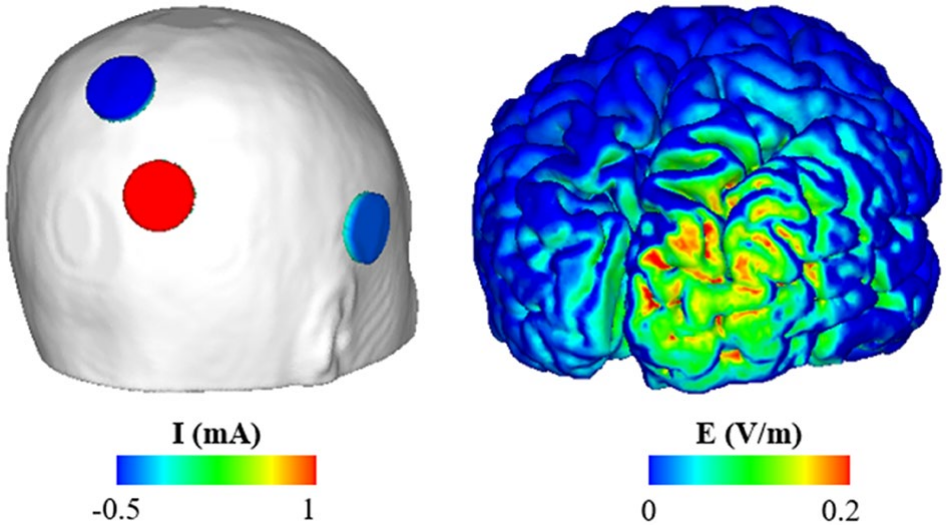
The final montage and electric field distribution. (From left to right) the three-channel electrode montage and optimal injection current amplitude used in this study and electric field distribution under the determined electrode condition.

The P3-PO4 montage, which resulted in the maximum electric field intensity over rIPS, was used to determine the final three-electrode montage. Three electrodes with the largest injection currents were Pz, PO4, and T8. The optimal injection current condition determined by additional LS revealed that the optimal injection current amplitudes were 1 mA, 0.64 mA, and 0.36 mA for PO4, Pz, and T8 electrodes, respectively. The maximum electric field over ROI for the final montage was 0.32 V/m, an increase of 0.09 V/m–0.13 V/m from maximum electric field amplitude when employing 15-channel montages. The final montage and the ROI are illustrated in Fig. 5. Also, the characteristics of electric field distribution under optimal electrode conditions and selected channels for each montage are shown in Table 1.

**Figure 6:**
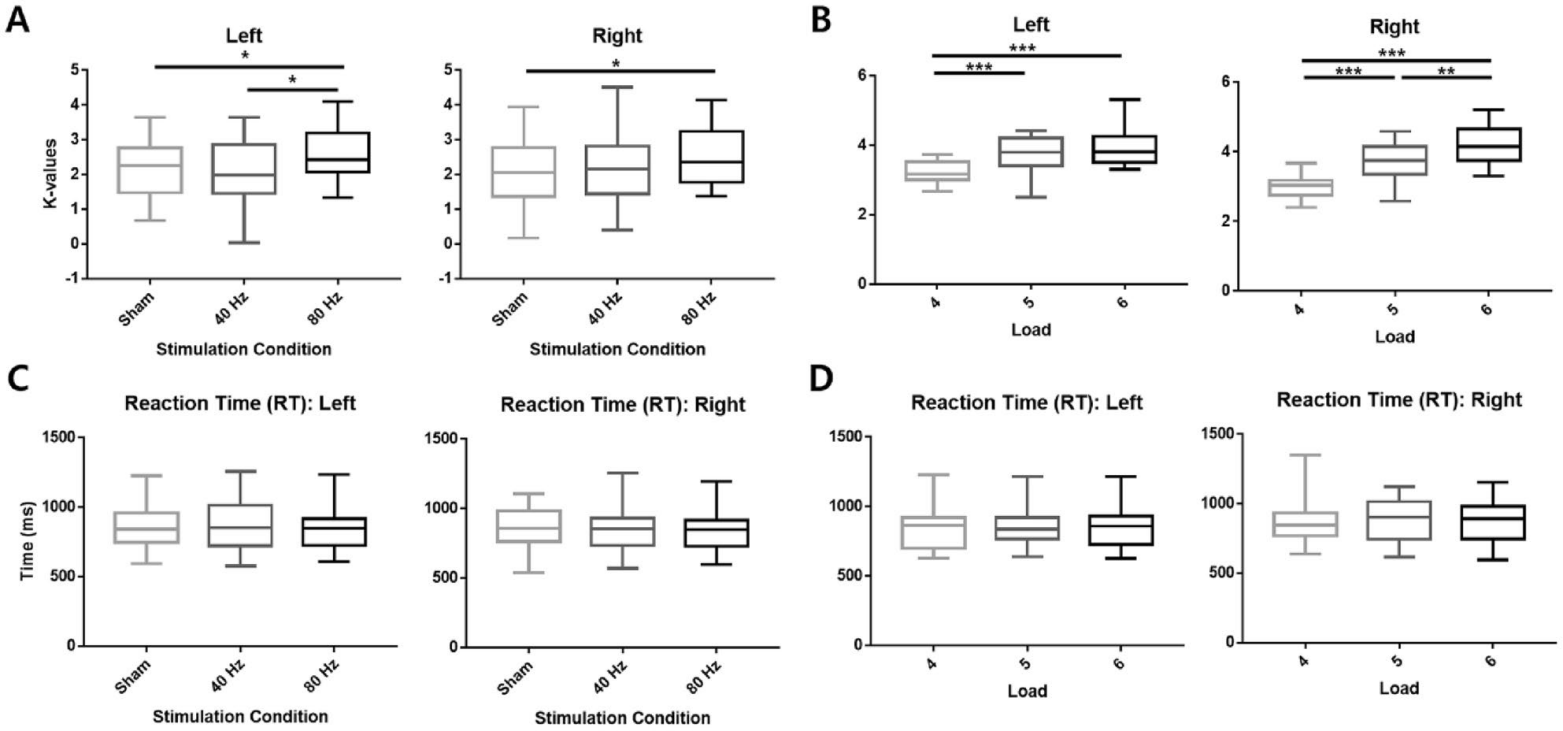
The behavioral performances under each stimulation condition. K-value and reaction time changes for the factors, condition and load. (**A**) Change of the K-values relative to the factor, condition, (**B**) change of the K-values relative to the factor, load, (**C**) change of the reaction time relative to the factor, condition, and (**D**) change of the reaction time relative to the factor, condition. K-values of both visual hemifields were affected by the factor, condition, increasing under 80 Hz stimulation compared to sham. The K-values also increased as the load increased, which again emphasizes that K-values are a quantitative representation of the factor, load. The reaction time was not affected by either condition or load. *p < 0.05; **p < 0.01; ***p < 0.001. All p-values are corrected values using FDR.

Behavioral results showed the main effect of the condition on both the left and right hemifields (left: χ^2^ = 6.1, *p* = 0.047, τ = 0.73; right: χ^2^ = 6.1, *p* = 0.047, τ = 0.73). The τ values represent effect sizes for the data. On the left hemifield, the *post-hoc* analysis with Wilcoxon’s signed-rank test indicated a significant enhancement of performance under 80 Hz stimulation condition, which was shown by the K-values between the 80 Hz vs. sham conditions and 80 Hz vs. 40 Hz conditions (80 Hz vs. sham: FDR corrected *p* = 0.027, 80 Hz vs. 40 Hz: FDR corrected *p* = 0.027). However, 40 Hz tACS did not show a significant change in the K-values compared to sham (*p* = 0.68). Furthermore, 80 Hz tACS exhibited a significant increase in the K-values of the right hemifield compared with the sham condition (*p* = 0.014), whereas no significant effect was observed in the other comparisons (80 Hz vs. 40 Hz: *p* = 0.15, 40 Hz vs. sham: *p* = 0.50). The results are shown in Fig. 6A, and the mean and standard deviation of K_L_ and K_R_ values under each stimulation condition are listed in Table 2.

**Table 2:** The mean and standard deviation of K_L_ and K_R_ values under each stimulation condition.

	Sham	40 Hz	80 Hz
K_L_	2.17 ± 0.83	1.9 ± 0.98	2.61 ± 0.8
K_R_	2.13 ± 1.0	2.0 ± 0.85	2.52 ± 0.84

The main effect of load on WM capacity was also observed in both hemifields (left: χ^2^ = 20.1, *p* < 0.001, τ = 0.67; right: χ^2^ = 29.2, *p* < 0.001, τ = 0.69). *Post-hoc* analysis showed significant differences among all loads on both hemifields (*p* < 0.002), except for that between loads five and six of the left hemifield (*p* = 0.23). As illustrated in Fig. 6B, the K-value tended to increase as the load increased; this was not unexpected as K-values reflect WM loads more than accuracy. Finally, Friedman’s test on the reaction time of both hemispheres indicated no significant effect for either factor, condition (right: *p* = 0.95, χ^2^ = 0.1, τ = 0.75; left: *p* = 0.86, χ^2^ = 0.3, τ = 0.8) or load (right: *p* = 0.14, χ^2^ = 4, τ = 0.91; left: *p* = 0.21, χ^2^ = 3.1, τ = 0.93). The analysis result of the reaction time is shown in Fig. 6C,D.

## Discussion

In this study, we observed differential effects of 80 Hz and 40 Hz tACS over rIPS on VWM performance, as tACS could affect neural oscillations differently depending on its stimulation frequency^28^. We targeted IPS as it is widely known that IPS plays a key role as a part of fronto-parietal network during VWM performance especially during maintenance of the presented items^29,30^. Our results demonstrated that working memory capacity increased regardless of visual hemifields when 80 Hz tACS was applied over rIPS. The K_L_ value increased by 0.44 and 0.71 under 80 Hz tACS compared to sham and 40 Hz tACS, respectively, and the K_R_ value increased by 0.39 and 0.52 under 80 Hz tACS compared to sham and 40 Hz tACS, respectively. While the increase capacity may not seem significant, it is an increase of 18.13%, at the very least (K_R_ of sham vs K_R_ of 80 Hz tACS).

Further analysis of reaction time showed no significant difference for both condition and load, underpinning that the stimulation protocol did not alter other components of cognitive function, such as speed of visual search or visual input processing. Additionally, the K-values under each load differed significantly in both hemispheres. Indeed, the K-values increased as the load increased, which seems obvious because K-values are the representative of the WM capacity required to complete the WM task. While no statistical difference was observed for loads four and five of the left hemifield, this is believed to be due to upper limit of individual WM capacity. It is reported that WM capacity is usually around 2–4^23^, and the mean of K-values observed for set size 5 and 6 of the left hemifield was 3.73 and 3.94, respectively. While K-values increased for set size of 5 and 6 for right visual hemifield, but the observed K-values were also around 4 (3.72 and 4.19 for set sizes 5 and 6, respectively). Additionally, we did not report statistical results using within factor Hemifield, as we believed analysis of hemifield specific K-value without accounting for stimulation conditions could be misleading; note that within factor load is absolutely unaffected by stimulation conditions, as load is only affected by the number of items presented during the task.

Although we hypothesized that 80 Hz tACS would enhance VWM performance and the opposite for 40 Hz, 40 Hz tACS did not alter VWM performance. Indeed, two possibilities may exist as to why: the first possibility is that although evidence is clear that low gamma activity (30–50 Hz) plays an important role in the WM process^31–33^, an increase in WM capacity might be more closely associated with an increase in high gamma activity (50–80 Hz)^34–36^; the second possibility is that 40 Hz may not be the lower bound of the gamma-band. Indeed, EEG may vary inter- and intra-individually^37,38^, and frequencies as low as 24–30 Hz are sometimes considered the lowest bound of the gamma-band^39–41^. Furthermore, some studies relating low gamma oscillation to WM performance have reported an increase of brain activities around 40 Hz^42,43^. For both reasons, it can be suggested with caution that 40 Hz stimulation might have kept the brain in a status quo with the sham condition and thus caused no significant difference in the behavioral performances. Employing low-frequency tACS to downregulate gamma oscillation^44,45^ or using 30 Hz tACS might have been more effective in decreasing WM capacity than applying 40 Hz tACS. Additionally, utilizing brain imaging methods would be necessary to further validate such mechanisms involving WM; indeed, integrative approach, including imaging methods, in neurostimulation experiments is suggested to be critical to define causal role of neural oscillations^46^. In the present study, we could not record EEG signals while the participants were performing behavioral tasks, as the tasks were performed simultaneously with the stimulation; EEG recorded simultaneously with the stimulations would distort the waveforms and might contain severe artifacts. It would be necessary to investigate the neural substrate of our tACS results with imaging methods in the future study. For example, comparing EEG features acquired before and after the stimulation sessions would provide helpful insight to understand the mechanism of tACS.

One notable observation was that 80 Hz tACS increased WM capacity in both left and right hemifield trials. In previous studies, only contralateral visual hemifield was affected by the facilitation of unilateral IPS^12,47^. Indeed, we initially expected that only the WM capacity of the left hemifield would be modulated by tACS over the rIPS. However, activation of the ipsilateral IPS is not uncommon when performing hemifield-specific VWM tasks^48,49^. The bilateral IPS is considered an integral part of the frontoparietal network, which is related to the perception and encoding of visual stimuli. Combined with reports that suggested that gamma oscillations play a critical role in the communication of brain regions, it appears that gamma tACS might facilitate such communication, eventually increasing the WM capacity in both hemispheres. Note that this is the first study to investigate the hemifield-specific effects of high gamma tACS, and thus further studies are still required to verify our hypotheses.

To avoid the stimulation of other brain regions, we developed and employed a multichannel montage specifically to stimulate the rIPS. By examining the electric field distribution by the proposed montage, we found that the stimulated cortical area was relatively focal compared with the previous studies with conventional montages^12,50–52^. It was also confirmed by the cortical electric field distributions that our results were not affected by the stimulation of brain areas other than the rIPS (i.e., the dorsolateral prefrontal cortex or retina). It is to be noted that no participant reported any kind of adverse effects (i.e., itchiness, phosphene, tingling sensations, etc.).

It is a concern, however, that whether our model represents the group of participants well. While it is optimal to obtain simulation results from each individual, using a sample head model in a multi-channel stimulation experiment is not uncommon^53–55^. The individual whose MRI was used in the simulation of the current study was young (with same range of age as the participants) and healthy male with the same nationality as the participants.

To the best of our knowledge, this is the first study to employ an unconventional stimulation frequency of 80 Hz over the IPS and report successful enhancement of VWM performance. Furthermore, we demonstrated that gamma tACS applied over a unilateral IPS could affect visuospatial working memory of both visual hemispheres. Based on these observations, it may be an interesting future topic to apply 80 Hz tACS rather than 40 Hz tACS to better modulate a variety of higher cognitive functions associated with theta-gamma CFC, such as motor control and multisensory integration^56^. It is also expected that the high gamma tACS first attempted in this study would be a promising method for neurorehabilitation aiming to enhance multiple higher cognitive functions.

There is a possibility that another stimulation frequency in the high gamma-band better enhances other cognitive functions. This should be considered in a future study. Additionally, it could be argued that targeting slow waves could be more effective in enhancing WM capacity because gamma oscillations occur in bursts while theta component of the PAC is observed more continuously. Therefore, a comparison between effects of tACS with different parameters could be another interesting future topic. Furthermore, while it appears that 80 Hz tACS strengthened communication between the bilateral IPSs, more studies are still required to confirm this effect with functional brain imaging methods such as EEG and fMRI. If it is demonstrated that 80 Hz tACS actually facilitates communications with different cortical regions, a multi-site tACS using a stimulation frequency in the high gamma band would be an interesting future topic.

## Conclusion

This study showed that WM capacity could be increased by applying high gamma tACS (80 Hz) over rIPS, while low gamma tACS (40 Hz) had no significant effect. Additionally, 80 Hz tACS proved to be effective on both visual hemifields, increasing the WM capacity of both the left and right visual hemifields. Our results suggest that high gamma tACS increased the number of items memorized during WM performance by entraining the intrinsic oscillation of rIPS to higher frequency; and also, such entrainment may have strengthened the communication between the left and right IPSs, as gamma oscillations are frequently observed between functionally connected regions. It is expected that our findings may provide useful insights for developing new therapeutic interventions for various neuropsychiatric disorders related to memory.

## Data Availability

The data and materials are conditionally available upon request. Please contact the corresponding author (ich@hanyang.ac.kr).

## Abbreviations

WM: Working memory
EEG: Electroencephalography
VWM: Visuospatial working memory
tDCS: Transcranial direct current stimulation
tACS: Transcranial alternating current stimulation
CFC: Cross frequency coupling
IPS: Intraparietal sulcus
rIPS: Right intraparietal sulcus
FEM: Finite element method
LS: Least squares
